# Development and validation of machine learning models with blood-based digital biomarkers for Alzheimer’s disease diagnosis: a multicohort diagnostic study

**DOI:** 10.1016/j.eclinm.2025.103142

**Published:** 2025-03-05

**Authors:** Bin Jiao, Ziyu Ouyang, Xuewen Xiao, Cong Zhang, Tianyan Xu, Qijie Yang, Yuan Zhu, Yiliang Liu, Xixi Liu, Yafang Zhou, Xinxin Liao, Shilin Luo, Beisha Tang, Zhigang Li, Lu Shen

**Affiliations:** a Department of Neurology, Xiangya Hospital, Central South University, Changsha, China; b National Clinical Research Center for Geriatric Disorders, Central South University, Changsha, China; c Engineering Research Center of Hunan Province in Cognitive Impairment Disorders, Central South University, Changsha, China; d Hunan International Scientific and Technological Cooperation Base of Neurodegenerative and Neurogenetic Diseases, Changsha, China; e Key Laboratory of Hunan Province in Neurodegenerative Disorders, Central South University, Changsha, China; f Brain Research Center, Central South University, Changsha, China; g FuRong Laboratory, Changsha, China; h Department of Geriatrics, Xiangya Hospital, Central South University, Changsha, China; i College of Information Science and Engineering, Northeastern University, Shenyang, China; j Hebei Key Laboratory of Micro-Nano Precision Optical Sensing and Measurement Technology, Qinhuangdao, China

**Keywords:** Blood-based digital biomarkers, Alzheimer’s disease, Machine learning, Diagnosis, Spectroscopy, Neurodegenerative diseases

## Abstract

**Background:**

Alzheimer’s disease (AD) involves complex alterations in biological pathways, making comprehensive blood biomarkers crucial for accurate and earlier diagnosis. However, the cost-effectiveness and operational complexity of method using blood-based biomarkers significantly limit its availability in clinical practice.

**Methods:**

We developed low-cost, convenient machine learning-based with digital biomarkers (MLDB) using plasma spectra data to detect AD or mild cognitive impairment (MCI) from healthy controls (HCs) and discriminate AD from different types of neurodegenerative diseases. Retrospective data were gathered for 1324 individuals, including 293 with amyloid beta positive AD, 151 with mild cognitive impairment (MCI), 106 with Lewy body dementia (DLB), 106 with frontotemporal dementia (FTD), 135 with progressive supranuclear palsy (PSP) and 533 healthy controls (HCs) between July 2017 and August 2023.

**Findings:**

Random forest classifier and feature selection procedures were used to select digital biomarkers. MLDB achieved area under the curves (AUCs) of 0.92 (AD vs. HC, Sensitivity 88.2%, specificity 84.1%), 0.89 (MCI vs. HC, Sensitivity 88.8%, specificity 86.4%), 0.83 (AD vs. DLB, Sensitivity 77.2%, specificity 74.6%), 0.80 (AD vs. FTD, sensitivity 74.2%, specificity 72.4%), and 0.93 (AD vs. PSP, sensitivity 76.1%, specificity 75.7%). Digital biomarkers distinguishing AD from HC were negatively correlated with plasma p-tau217 (*r* = −0.22, *p* < 0.05) and glial fibrillary acidic protein (GFAP) (*r* = −0.09, *p* < 0.05).

**Interpretation:**

The ATR-FTIR (Attenuated Total Reflectance-Fourier Transform Infrared) plasma spectra features can identify AD-related pathological changes. These spectral features serve as digital biomarkers, providing valuable support in the early screening and diagnosis of AD.

**Funding:**

The 10.13039/501100001809National Natural Science Foundation of China, STI2030-Major Projects, 10.13039/501100012166National Key R&D Program of China, Outstanding Youth Fund of 10.13039/501100004735Hunan Provincial Natural Science Foundation, Hunan Health Commission Grant, Science and Technology Major Project of Hunan Province, Hunan Innovative Province Construction Project, Grant of National Clinical Research Center for Geriatric Disorders, 10.13039/501100011790Xiangya Hospital and Postdoctoral Fellowship Program of CPSF.

**Research in context**

**Evidence before this study**

Previous studies have shown the feasibility of identifying Alzheimer’s disease (AD) using a blood-based multi-biomarker panel and machine learning techniques. However, it is not practical to implement on a population level due to the limitation of cost, complexity, sensitivity, specificity, reliability and reproducibility of blood-based biomarkers measurements. Digital biomarkers offer a transformative approach to disease diagnosis and management, particularly for complex conditions like AD. Thus, a method using blood-based digital markers that facilitates large-scale pre-screening and early diagnosis is desired. We conducted a comprehensive search in PubMed, medRxiv, bioRxiv, and arXiv for English-language articles published from inception to Oct 6th, 2024, using the terms “((“blood-based Alzheimer’s disease biomarkers”) OR (“ATR_FTIR” OR “FTIR”) OR (“Raman”) OR (“digital ELISA” OR “Simoa”) OR (“IP-MS“) OR (“MSD”) OR (“Machine learning”))”. The transformation of traditional biochemical plasma markers into digital biomarker is mostly achieved by digital Single-Molecule Array (Simoa), Immunoprecipitation-mass spectrometry (IP-MS) and Meso Scale Discovery platform (MSD). Only a very small part of the conversion is achieved by Fourier Transform Infrared (FTIR) or Raman spectroscopy test platforms. Combined with machine learning techniques, spectroscopy test platform as reagent-free, non-destructive approach has been used in diagnosis and screening. At the same time, fewer pilot studies have used machine learning algorithms combined with spectral techniques to diagnose dementia. Until now, machine-learning methods for diagnosing AD with interpretable spectral digital blood-based biomarkers remains poorly investigated.

**Added value of this study**

Our findings provide evidence for the feasibility of large-scale screening and early diagnosis using a machine-learning system with blood-based digital biomarkers. Unlike digital biomarkers that focus on smartphones and wearables, our method can transform conventional plasma biomarkers into digital biomarkers by spectral techniques and construct machine learning-based models with these digital biomarkers panel for enhanced predictive capabilities. This novel framework supports multi-omics integration, providing a more comprehensive view of disease biology. The digital biomarkers generated are particularly suited for large-scale population screening and early diagnosis, bridging the gap between clinical relevance and technological advancements.

**Implications of all the available evidence**

This study built stable, mini-invasive and easily accessible diagnostic models to accelerate the path to preventive intervention and diagnosis of AD and mild cognitive impairment (MCI). Our study highlights the potential of blood-based digital biomarkers to develop streamlined machine learning diagnostic models, enhancing large-scale screening and early diagnosis. The digitization of these biomarkers enables their integration into digital health technologies, potentially allowing for diagnostic and monitoring purposes in precision medicine. The convergence of blood spectral test technologies and machine learning approach presents a valuable opportunity for developing advancing disease prediction strategies, which could drive future research. and improve patient outcomes through better predictive strategies.

## Introduction

Alzheimer’s disease (AD) is the most common form of dementia, imposing a heavy burden on the public and health care systems.^1^ Early diagnosis of AD is crucial for timely interventions and disease-modifying treatments. Currently, AD diagnosis involves measuring amyloid beta (Aβ) and phosphorylated tau (p-tau) protein levels using cerebrospinal fluid (CSF) and positron emission tomography (PET) examinations.^2^^,^^3^ These approaches are costly, invasive, and highly relies on clinical infrastructure, thus significantly limiting its availability in clinical practice. Minimally invasive blood-based biomarkers as new AD biomarker candidates have, therefore, been used to identify biochemical pathway alterations linked to AD and provide a more complete picture of the metabolomic basis of dementia pathology. Blood-based markers (BBM) have been added to the revised criteria for diagnosis and staging of AD proposed by Alzheimer’s Association Workgroup (AA).^4^ The complex pathophysiological processes of AD require a novel, more holistic approach to analysis, rather than trying to identify individual “biomarkers”—much like the comprehensive strategies already embraced in fields such as genomics, proteomics, and metabolomics. However, the utilization of high-performance plasma biomarker panels remains costly and complex, which hinders their use as widespread screening tools and first-line diagnostics.

In the field of biomedical applications, integration of the field of vibrational spectroscopic analysis with bioinformatics analysis has been recognized as the key to realizing its true potential and called spectralomics.^5^ By analogy, Fourier transform infrared (FTIR) spectroscopy, as a label-free optical biosensor, can be considered a form of “spectralomics”, generating a distinctive digital fingerprint and delivers a high content, holistic characterization of biological samples. By harnessing the interaction of infrared light with biological matter, it simultaneously examines functional groups, bonding types, and molecular conformations associated with various compounds, including proteins, amino acids, lipids, carbohydrates, and nucleic acids. This technology is particularly adept at translating chemical and structural changes at the molecular level, which are induced by diseases and pathological conditions, into discernible blood plasma spectra digital biomarkers.^6^, ^7^, ^8^ Spectral digital markers optimized by feature selection methods in machine learning can offer a more robust and reliable method of capturing the biochemical changes induced by pathologies.^5^ Currently, ensuring transparency and interpretability in diagnostic decision-making remains a key focus in the development of machine learning-based models.^9^ In our study, analyzing the correlation between the peaks of spectral digital biomarkers and the concentration of pathological biomarkers enhances the clarity of diagnostic outputs, allowing for more precise and interpretable models that can be more easily integrated into clinical practice. Based on panels containing optimized blood-based spectra biomarkers, digitally facilitated diagnostic models hold great promise to transform the diagnostic and prognostic evaluation of AD.

In our research, we utilized FTIR spectroscopy to analyze plasma samples collected from 1324 individuals. Additionally, we employed single molecule immune detection (SMID) technology to measure the levels of plasma p-tau217, Aβ42, and GFAP in the participants. In summary, the aims of this study were to develop innovative blood-based digital biomarker panels, assess their efficacy in AD diagnose, early diagnose and differential diagnosis, and evaluate the relationship between AD digital biomarkers and plasma biomarkers to provide biological interpretation for digital panels.

## Methods

### Participants

Four independent cohorts were included in this study. The discovery cohort (Cohort 1) enrolled 464 participants, comprising 275 patients with AD and 189 HCs. The validation cohort (Cohort 2) from the Liuyang Community included 362 participants, with 18 patients with AD and 344 HCs. The pre-dementia cohort (Cohort 3) consisted of 151 patients with MCI. The differential diagnosis cohort (Cohort 4) comprised 347 patients with other neurodegenerative diseases, including 106 with FTD, 106 with DLB, and 135 with PSP.

All patients with AD from Cohort 1 and 2 were diagnosed based on the National Institute on Aging and Alzheimer’s Association criteria (2011),^10^ and all were Aβ positive. Among them, 200 patients underwent lumbar puncture and, 93 patients completed Pittsburgh compound B positron emission tomography (PiB-PET) examinations. FTD, DLB, and PSP were diagnosed according to their respective clinical criteria.^11^, ^12^, ^13^ Participants in Cohorts 1, 3, and 4 were enrolled from the Department of Neurology, Xiangya Hospital, Central South University, while those in Cohort 2 were from the Liuyang community. Enrollment occurring between July 2017 and August 2023. All HCs were recruited from the Liuyang community and showed no cognitive decline after two years of follow-up. All participants in this study are of Han Chinese ethnicity.

### Ethics

This study was approved by the ethical review committee of Xiangya Hospital, Central South University (No. 2022020483), and conducted in accordance with the tenets of the Declaration of Helsinki. All participants provided written informed consent.

### Neuropsychological assessment

All patients underwent a comprehensive battery of neuropsychological tests, including the MMSE, Montreal Cognitive Assessment (MoCA), and Clinical Dementia Rating (CDR). All HCs completed the MMSE assessment.

### PiB-PET acquisition and interpretation

PiB-PET imaging was performed and analyzed as previously described.^14^ Images were acquired 50 min after intravenous injection of PiB (12 mCi over 20 s). Radiotracer accumulation in the lateral temporal and frontal lobes, posterior cingulate cortex/precuneus, and parietal lobes was used to assess Aβ deposition, using the cerebellum as the reference region. Results were interpreted by three nuclear medicine physicians. Standardized uptake value ratios (SUVR) were calculated by comparing regional PiB retention to cerebellar grey matter. Individuals with SUVR values exceeding 1.5 in any region of interest (ROI) were classified as Aβ-positive.

### Cerebrospinal fluid collection and analysis

CSF was collected via lumbar puncture, centrifuged at 2000×*g* at 4 °C for 10 min, and stored at −80 °C. All CSF biomarkers were measured by enzyme-linked immunosorbent assay (ELISA), including Aβ40 (EQ 6511-9601-L), Aβ42 (EQ 6521-9601-L), t-tau (EQ 6531-9601-L), and p-tau181 (EQ 6591-9601-L) (EUROIMUN, Germany). According to the manufacturer’s instructions, participants with Aβ42 < 550 pg/ml or Aβ42:551–650 pg/ml along with Aβ42/40 ≤ 0.1 were classified as Aβ-positive (A+), and participants with p-tau ≥ 61 pg/ml were classified as tau-positive (T+).

### Sample collection and processing

Venous blood samples were collected in ethylenediaminetetraacetic acid (EDTA) tubes and centrifuged at 3000 rpm for 15 min at 4 °C within 2 h of collection. The obtained blood samples for plasma were stored at −80 °C and were not subjected to freeze-thaw cycles prior to use. P-tau217, GFAP, and Aβ42 were quantified on a fully automated SMID machine (AST-Sc-Lite; AstraBio, Suzhou, China) according to the manufacturer’s instructions. P-tau217 (R64050), GFAP (R64060), and Aβ42 (R64020) assay kits (AstraBio) were used for detection. Surgeons were blinded to the group status of the participants.

### Spectral data acquisition and processing of plasma sample

Spectra of plasma samples were obtained using an Alpha FTIR spectrometer with attenuated total reflection (ATR) attachment (Bruker Optics Ltd.), operated with OPUS 5.5 software. Plasma samples (2 μL) were deposited on a KBr slide and air-dried for 12 min. An auto-presser applied uniform pressure to ensure reproducible measurements. Spectra were scanned 32 times in the range of 4000–950 cm^−1^ at 4 cm^−1^ resolution. The diamond crystal was cleaned before each sample analysis, and a background spectrum was obtained after each scan to account for environmental variations (Supplementary Fig. S1).

Derivative spectroscopy is an established technique for resolving overlapping spectra and accounting for differences in more detail. The Savitzky–Golay (SG) algorithm, a landmark development in derivative spectroscopy, was employed in this study. The second derivative spectra of blood plasma samples were obtained using SG (second-order polynomial and nine filter coefficients).

### Spectral data detection of p-tau, GFAP, and Aβ42

Human p-tau (TAU-H5147) and GFAP (GFP-H5143) were supplied by the Acrobio Systems Group. Human Aβ42 protein (ab120301) was purchased from ABCAM. For testing, they were dissolved at room temperature and diluted 10-fold. After thawing and dilution, 10 μL from each sample was deposited on the ATR sampling area. The ATR-FTIR spectra of the protein samples were scanned 32 times and recorded in the range of 4000–600 cm^−1^ at a 4 cm^−1^ resolution. An air background spectrum was used to reduce the impact of environmental changes.

### Diagnostic model with blood-based digital biomarkers panel

Kennard-Stone (KS) sample set partitioning, Relief-F feature selection and RF classifier were used to develop diagnostic models.^15^, ^16^, ^17^ The following steps were taken to develop the AD diagnostic model using Cohort 1:

(1) Participants were divided into discovery, validation, and test sets using KS partitioning in a 7:1.5:1.5 ratio;
(2) Spectral marker importance was ranked using Relief-F feature selection;
(3) An RF classifier was trained on the discovery cohort as the primary diagnostic model;
(4) Spectral markers were iteratively extracted until sensitivity and specificity exceeded 80%;
(5) Pathogenic protein digital markers were identified based on p-Tau, Aβ42, and GFAP absorption peaks;
(6) Spectral markers common to both machine learning-based preselected biomarkers and pathogenic protein markers were selected for the AD diagnosis panel;
(7) The final RF diagnostic model using the spectral biomarker panel achieved high-accuracy AD diagnosis.

Models for distinguishing MCI from HCs, AD from MCI, and AD from other neurodegenerative diseases were constructed similarly, with their respective spectral biomarker panels.

### Statistical analysis

Model performance was evaluated by comparing detection results with clinical diagnoses. Specificity and sensitivity were calculated to evaluate diagnostic performance. Sensitivity, specificity, and receiver operating characteristic (ROC) curves were used to assess performance, with area under the curve (AUC) values calculated. Both processing and computational analyses for constructing the diagnostic model were performed using MATLAB R2016a software (MathWorks). Descriptive statistics were reported as frequencies or medians. The Mann–Whitney U test and chi-square test were used to compare continuous and categorical variables, respectively. DeLong test was performed to compared AUCs. Linear regression assessed the relationship between spectral biomarkers and MMSE scores or plasma biomarker levels. Plasma marker levels were logarithmic transformed to make their distribution more consistent with a normal distribution. All statistical analyses were performed using Python version 3.8.10. Statistical significance was set at *p* < 0.05. All tests were two-tailed.

### Classification model evaluation

Several traditional classification models were compared through pre-experimental research in Cohort1, including random forest (RF), support vector machine (SVM), K-Nearest Neighbors (KNN), Logistic Regression (LR), Linear discriminant analysis (LDA) and Back Propagation Neural Network (BP). The performance of these models was evaluated in both the training cohort and validation cohort, focusing on sensitivity and specificity.

### Role of funding source

The research fundings for the study did not influence the study design, data gathering, data processing, data interpretation, or the composition of the manuscript.

## Results

### Baseline characteristics

This study included 1324 participants from four independent cohorts. The discovery cohort (Cohort 1) included 275 patients with AD and 189 healthy controls (HCs). The validation cohort (Cohort 2), drawn from the community, included 18 patients with AD and 344 HCs. The pre-dementia cohort (Cohort 3) included 151 patients with MCI. The differential diagnosis cohort (Cohort 4) included 106 patients with dementia with Lewy bodies (DLB), 106 with frontotemporal dementia (FTD), and 135 with progressive supranuclear palsy (PSP). Table 1 summarizes baseline demographics, cognitive assessments, CSF biomarkers, and plasma biomarker information. Plasma biomarkers included p-tau217, Aβ42, and GFAP. All AD patients from Cohort 1 and Cohort 2 were Aβ+ positive, confirmed through CSF (n = 200) and Aβ-PET (n = 93) examinations. Plasma p-tau217 and GFAP levels were significantly higher in AD patients (*p* < 0.05), while Aβ42 levels were significantly lower compared to HCs (*p* < 0.05). Significant differences in MMSE scores were observed between patients with MCI, DLB, FTD, PSP and HC (*p* < 0.05, Supplementary Table S1).

**Table 1:** Characteristics of 1324 participants enrolled in this study.

	Cohort 1 (n = 464)			Cohort 2 (n = 362)			Cohort 3 (n = 151)	Cohort 4 (n = 347)		
	AD (n = 275)	HC (n = 189)	*p*	AD (n = 18)	HC (n = 344)	*p*	MCI (n = 151)	DLB (n = 106)	FTD (n = 106)	PSP (n = 135)
Age, years	65 [55–72]	68 [65–71]	<0.001	59 [55–65]	67 [64–71]	<0.01	67 [61–72]	72 [66–78]	63 [55–69]	66 [60–70]
Sex, Male	114 (41.5)	88 (46.6)	0.3	8 (44.4)	168 (48.8)	0.7	71 (47)	63 (59.4)	55 (51.9)	88 (65.2)
ADO, years	60 [53–69]	–		57 [52–61]	–		63 [57–68]	69.5 [62–75]	58 [53–66]	63 [57–68]
COD, years	2 [1–4]	–		2.5 [2–4]	–		2 [1–3]	2 [1–4]	2 [1–4]	2 [1.5–4]
Education, years	9 [6–12]	9 [6–12]	0.044	9 [9–12]	6 [4–9]	<0.01	9 [6–12]	9 [6–12]	9 [6–12]	9 [6–12]
MMSE	15 [8–20]	29 [29–30]	<0.001	15 [9.5–21]	27 [25–28]	<0.01	25 [22–27]	15 [7–22]	16 [8–22]	24 [18–27]
MoCA	9 [4–14]	–	–	7.5 [4–15]	–	–	20 [16–21]	7 [3–13]	10 [4–15]	14 [10–19]
CDR	1 [0.5–2]	–	–	1 [0.5–2]	–	–	0.5 [0–0.5]	2 [1–2]	1 [0.5–2]	–
CSF Aβ42, pg/ml	359.34 [252.14–459.12]	–	–	488.88 [434.22–528.65]	–	–	–	–	–	–
CSF Aβ40, pg/ml	5807.83 [3621.25–9212.62]	–	–	7601.18 [4765.38–11275.75]	–	–	–	–	–	–
CSF Aβ42/40	0.06 [0.04–0.08]	–	–	0.07 [0.04–0.09]	–	–	–	–	–	–
CSF p-tau181, pg/ml	88.6 [48.64–133.98]	–	–	84.28 [73.57–124.24]	–	–	–	–	–	–
CSF t-tau, pg/ml	314.5 [198.34–559.96]	–	–	307.39 [57.85–479.01]	–	–	–	–	–	–
Plasma p-tau217 pg/ml	5.23 [4.2–6.76]	4.48 [3.6–6.05]	0.002	–	–	–	3.5 [3–4.01]	–	–	–
Plasma Aβ42 pg/ml	60.41 [48.06–85.75]	70.13 [52.11–92.7]	0.035	–	–	–	69.05 [47.74–98.18]	–	–	–
Plasma GFAP pg/ml	41.32 [27.92–61.16]	19.15 [14.92–27.91]	<0.001	–	–	–	24.67 [16.62–41.5]	–	–	–

Data are median [interquartile range] or n (%).

ADO, Age of disease onset; COD: course of disease; MMSE, Mini-Mental State Examination; MoCA, Montreal Cognitive Assessment; CDR, Clinical Dementia Rating Scale; AD, Alzheimer’s disease; HC, healthy control; MCI, mild cognitive impairment; DLB, Lewy Body dementia; FTD, frontal-temporal dementia; PSP, progressive supranuclear palsy; CSF, cerebrospinal fluid; GFAP, glial fibrillary acidic protein.

### AD diagnostic modelling

**Fig. 1:**
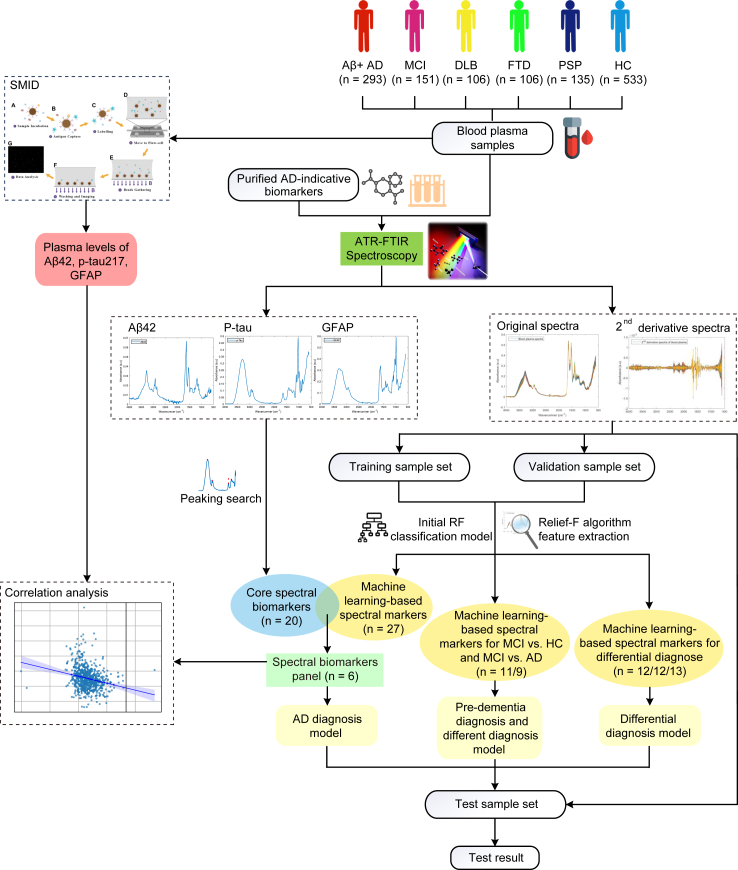
**Study design and diagnostic models’ construction flow.** AD, Alzheimer’s disease; MCI, mild cognitive impairment; DLB, Lewy Body dementia; FTD, frontal-temporal dementia; PSP, progressive supranuclear palsy; HC, healthy control; ATR-FTIR, attenuated total reflection Fourier transform infrared; GFAP, glial fibrillary acidic protein; RF, random forest.

**Fig. 2:**
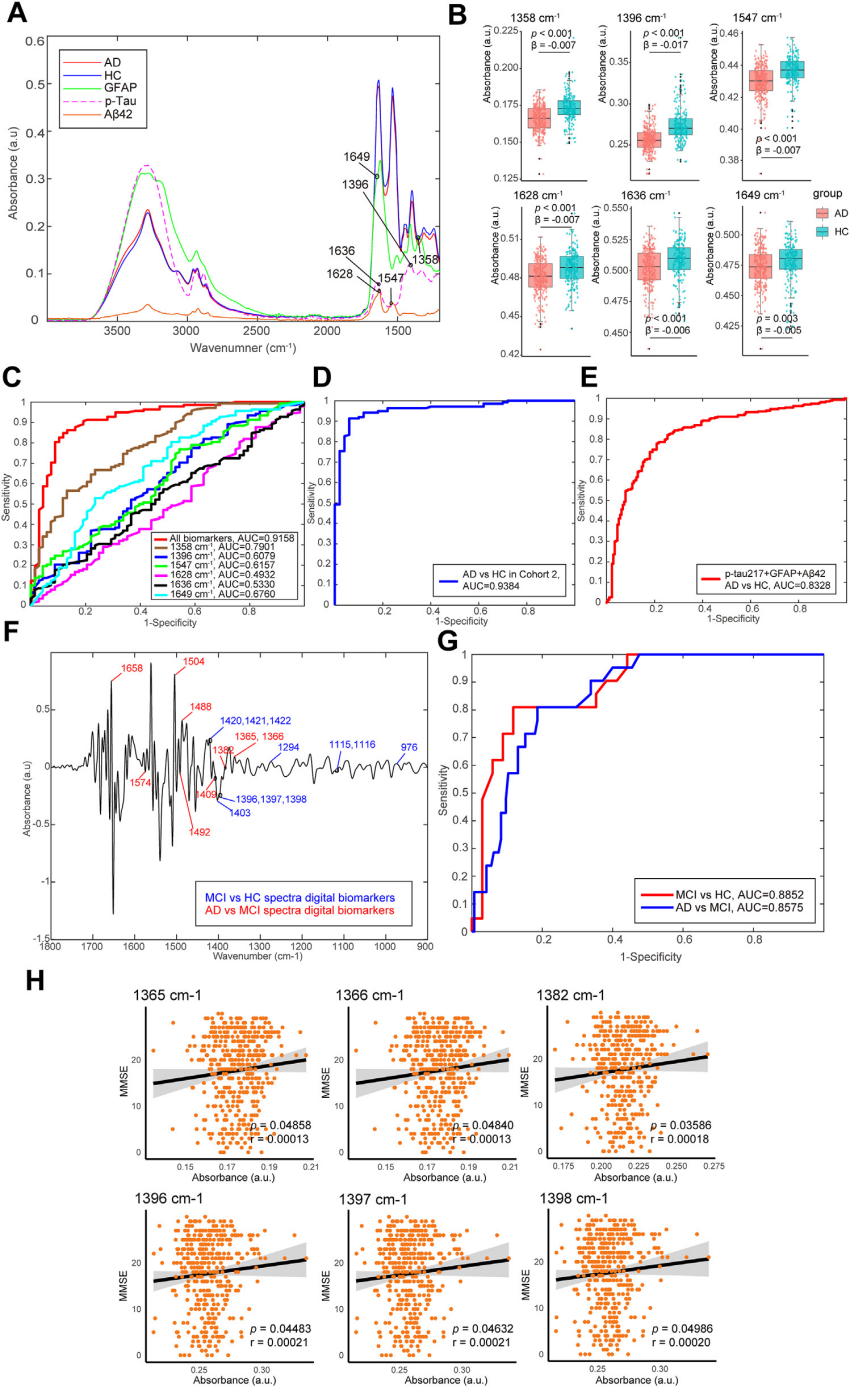
**ATR-FTIR spectra comparison and diagnostic performance between AD, MCI and HC participants.** (A) ATR-FTIR average spectra of patients with AD and HCs in Cohort 1 and the ATR-FTIR spectra of purified GFAP, p-Tau, Aβ42; AD spectra digital biomarkers were marked on the spectra of corresponding AD indicator biomarkers; (B) Box diagram of the six AD spectra digital biomarkers with significantly different levels between AD and HC participants in Cohort 1 (*p* < 0.05); (C) ROC curve of the discrimination power of each AD spectra digital biomarker and all six AD spectra digital biomarkers on the test set in Cohort 1; (D) ROC curve of diagnostic performance for AD vs. HC in Cohort 2; (E) ROC curve of the discrimination power of plasma biomarkers for AD vs. HC in Cohort 1; (F) Second derivative spectra of MCI patients in Cohort 3 and AD patients/HCs in Cohort 1, spectral digital biomarkers for distinguishing MCI and HC and for distinguishing MCI and AD were marked on the second derivative spectra with different colors; (G) ROC curve of performance for distinguishing MCI from HC and AD participants in Cohort 1; (H) Relationship between spectra digital biomarkers for distinguishing MCI and MMSE scores in AD of Cohort 1 and Cohort 3. AD, Alzheimer’s disease; HC, healthy control; GFAP, glial fibrillary acidic protein; AUC, area under curve; MCI, mild cognitive impairment; MMSE, mini-mental state examination; ROC, receiver operating characteristic.

The study design and data analysis process for constructing diagnostic models MLDB were shown in Fig. 1. The spectra of blood plasma from the training and validation sets were used to select twenty-seven machine learning-based spectral biomarkers associated with AD selected via the Relief-F feature extraction algorithm and the initial Random Forest (RF) classification model (Supplementary Table S2). The biochemical assignments for the machine learning-based spectral biomarkers were shown in Supplementary Table S3. Subsequently, the spectra of purified indicative biomarkers, including Aβ42, p-tau, and GFAP, were measured and compared with the plasma spectra of patients with AD to screen out twenty core spectral biomarkers, which were corresponding to or adjacent to the absorption peaks (Supplementary Table S2). From the intersection of machine learning-based spectral features and core spectra digital biomarkers, six AD spectra digital biomarkers were selected (Fig. 2A). These included absorption peaks at 1547 cm^−1^ and 1628 cm^−1^ for Aβ42; 1396 cm^−1^ and 1636 cm^−1^ for p-tau; and 1358 cm^−1^ and 1649 cm^−1^ for GFAP (Fig. 2A). These biomarkers were input into a RF algorithm to build an AD diagnostic model.

The absorbance of the average spectrum and all six AD spectral digital biomarkers in digital panel of patients with AD was lower than that of HCs (*p* < 0.05; Fig. 2A and B). Although neither single distinctive absorption peak exhibited good diagnostic efficiency, the combined panel of the six biomarkers achieved a sensitivity of 88.2%, a specificity of 84.1%, and an AUC of 0.92 for distinguishing AD from HCs (Fig. 2C). To further evaluate the diagnostic performance of AD digital panel, we tested Cohort 2 from the community. Diagnostic model achieved a sensitivity of 88.8%, a specificity of 86.4%, and an AUC of 0.94 for distinguishing AD from HC (Fig. 2D).

Comparatively, using plasma p-tau217, GFAP, and Aβ42 as biomarkers resulted in an AUC of 0.83, indicating the spectral biomarker panel performed better (*p* < 0.05) (Fig. 2E).

We conducted a performance evaluation of six classification models, including RF, SVM, LR, KNN, LDA and BP. The results of the classification model evaluation, presented in Supplementary Table S4, indicate that the Random Forest model consistently outperforms the other algorithms in terms of sensitivity and specificity. Additionally, we utilized both validation set and test set to evaluate the models, which helps prevent overfitting. This approach allows us to fine-tune the model using the validation cohort while keeping the test cohort completely separate to ensure that our performance metrics are reliable and generalizable to unseen data. We assessed the sensitivity and specificity of the models using both the validation and test sets. The consistent performance of these metrics across both sets indicates that the model is not overfitting.

### MCI diagnostic modelling

It is well-accepted that mild cognitive impairment (MCI) is a high-risk factor for the development of AD and reflects a prodromal predementia state of AD. In our study, eleven machine learning-based spectral biomarkers distinguishing MCI from HCs were selected via the Relief-F feature extraction algorithm and the initial RF classification model (Fig. 2F). Testing the model on MCI test samples in Cohort 3 and HCs test sample in Cohort 1 yielded a sensitivity of 88.8%, a specificity of 86.4%, and an AUC of 0.89 for distinguishing MCI from HCs.

Nine machine learning-based spectral biomarkers distinguishing MCI from AD were selected to make up its spectral biomarker panel (Fig. 2F). The MCI and AD discrimination model with corresponding spectral biomarkers panel showed a sensitivity of 75.8%, a specificity of 72.6%, and an AUC of 0.86 (Fig. 2G).

Six of the 20 biomarkers used to diagnose MCI (1365 cm^−1^, 1366 cm^−1^, 1382 cm^−1^, 1396 cm^−1^, 1397 cm^−1^, and 1398 cm^−1^) were positively correlated with Mini-Mental State Examination (MMSE) scores (Fig. 2H), suggesting their potential for tracking disease progression.

### Differential diagnostic modelling

**Fig. 3:**
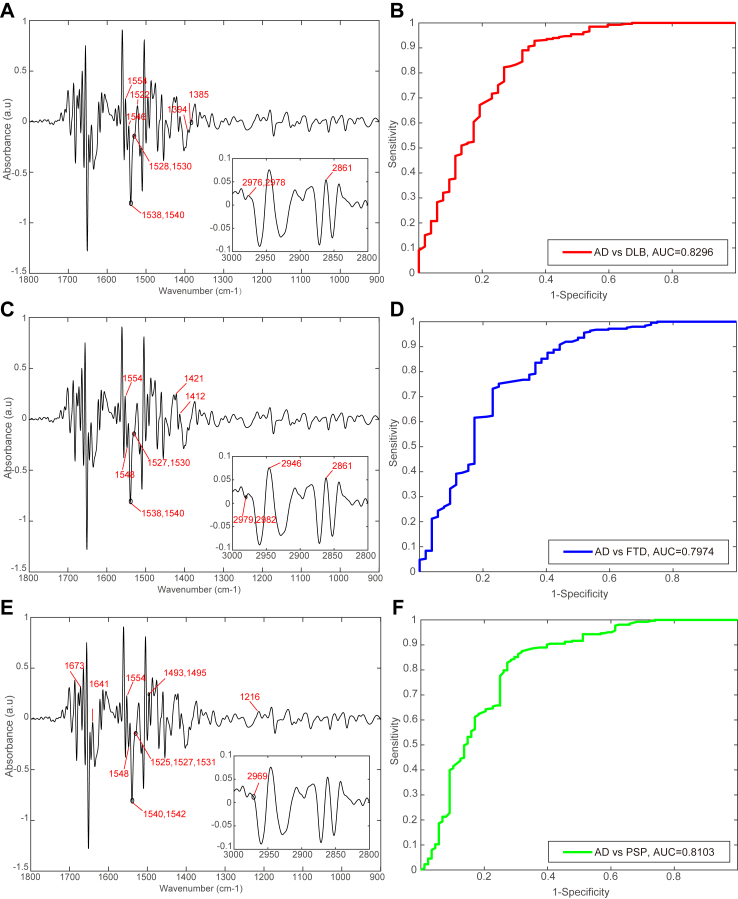
**Differential diagnostic performance of corresponding digital panel.** (A) Second derivative spectra of DLB patients in Cohort 4 and AD patients in Cohort 1, spectral digital biomarkers for distinguishing DLB and AD were marked on the second derivative spectra; (B) ROC curve of differential diagnostic performance for distinguishing DLB from AD; (C) Second derivative spectra of FTD patients in Cohort 4 and AD patients in Cohort 1, spectral digital biomarkers for distinguishing FTD and AD were marked on the second derivative spectra; (D) ROC curve of differential diagnostic performance for distinguishing FTD from AD; (E) Second derivative spectra of PSP patients in Cohort 4 and AD patients in Cohort 1, spectral digital biomarkers for distinguishing PSP and AD were marked on the second derivative spectra; (F) ROC curve of differential diagnose performance for distinguishing PSP from AD. AD, Alzheimer’s disease; DLB, Lewy Body dementia; FTD, frontal-temporal dementia; PSP, progressive supranuclear palsy; ROC, receiver operating characteristic.

**Fig. 4:**
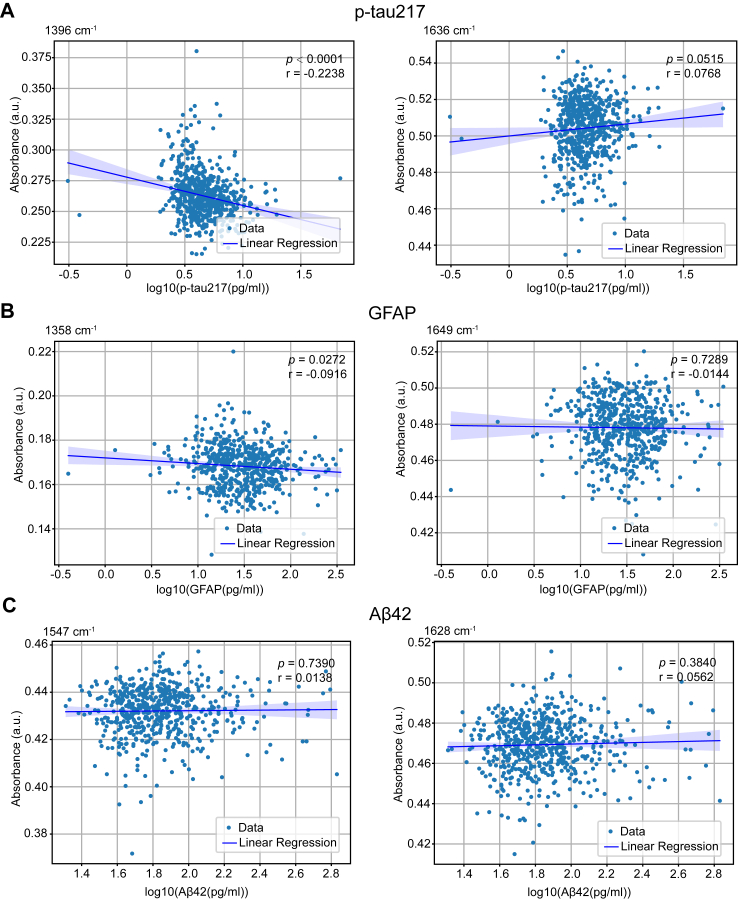
**A linear relationship between plasma GFAP, p-Tau and Aβ42 levels with absorbance of corresponding AD spectral biomarkers in patients with AD.** (A) Linear relationship between plasma p-Tau 217 levels and corresponding AD spectral biomarkers; (B) Linear relationship between plasma GFAP levels and corresponding AD spectral biomarkers; (C) Linear relationship between plasma Aβ42 levels and corresponding AD spectral biomarkers. GFAP, glial fibrillary acidic protein; AD, Alzheimer’s disease.

Twelve, thirteen and twelve machine learning-based spectral biomarkers respectively associated with DLB, FTD and PSP were selected to form corresponding spectral biomarkers panels (Fig. 3A, C, E). The AD and DLB differential diagnostic model achieved a sensitivity of 77.2%, a specificity of 74.6%, and an AUC of 0.83 (Fig. 4B). The AD and DLB differential model achieved a sensitivity of 74.2%, specificity of 72.4%, and AUC of 0.80 (Fig. 4D). The AD and PSP differential diagnostic model showed a sensitivity of 76.1%, a specificity of 75.7%, and an AUC of 0.81 (Fig. 3E and F).

### Relationship between spectral digital biomarkers and plasma biomarkers

To confirm the efficacy of digital panel in detecting AD, we measured plasma p-tau217, Aβ42, and GFAP levels in participants from Cohort 1 and Cohort 3. We conducted correlation analyses between the absorbance of the spectral digital biomarkers and the corresponding plasma biomarker levels. Absorption peaks at 1396 cm^−1^ and 1636 cm^−1^ associated with purified p-tau, showed that 1396 cm^−1^ was significantly negatively correlated with plasma p-tau217 (*r* = −0.22, *p* < 0.01) (Fig. 4A). Peaks at 1358 cm^−1^ and 1649 cm^−1^, associated with purified GFAP, showed that 1358 cm^−1^ was significantly negatively correlated with plasma GFAP (*r* = −0.09, *p* = 0.03) (Fig. 4B). Peaks at 1547 cm^−1^ and 1628 cm^−1^ associated with purified Aβ42, were not related to plasma Aβ42 (Fig. 4C).

## Discussion

For the first time, we explored the utility of blood-based spectral biomarkers panels as diagnostic tools for AD, and assessed their efficacy in a large Chinese cohort of 1324 individuals. We identified six AD-specific spectral biomarkers based on purified p-tau, Aβ42, and GFAP synthetic products, constructing an AD biomarker panel. The panel demonstrated high diagnostic accuracy with an AUC of 0.92, which was further validated in an independent cohort (AUC = 0.94). The model distinguished MCI from HC and AD with AUCs of 0.89 and 0.86, respectively. Additionally, the panel effectively differentiated other neurodegenerative dementias, such as DLB, FTD, and PSP from AD, with AUCs of 0.83, 0.80, and 0.81, respectively. We also observed a negative correlation between plasma p-tau217 and GFAP levels and AD spectral biomarkers, further supporting the diagnostic potential of our approach.

Blood-based biomarkers have garnered interest as potential diagnostic tools for AD. However, due to the complex pathology of AD, single biomarkers may not capture the full spectrum of pathological changes. Traditional molecular detection methods such as ELISA tests target individual molecules, limiting their capacity to reflect the disease’s multifactorial nature. In contrast, plasma FTIR-based spectral panels analyze molecular structures and chemical compositions related to the disease, allowing for improved identification of complex diseases.^18^ While individual spectral peaks may not be specific to AD, the composite panel forms a disease-specific “fingerprint” that enhances diagnostic accuracy. Similar to other omics approaches, like proteomics and metabolomics, spectralomics integrates multiple signals to represent underlying molecular changes, offering a holistic view of biological systems.^5^ Our study showed that combining multiple spectral peaks achieved higher diagnostic performance than individual peaks, supporting the utility of an omics-based approach in AD diagnostics.

The spectral regions identified in this study provide insights into the biochemical alterations associated with AD. The absorption peak at 1800-1700 cm^−1^, attributed to lipid C=O stretching, suggests lipid metabolism dysregulation, a factor implicated in neurodegeneration. Amide I and II bands (1750-1200 cm^−1^) indicate the presence of protein aggregates, such as Aβ fibrils, while shifts in these bands reflect protein conformational changes.^19^ The 1200-900 cm^−1^ region, linked to lipid peroxidation and oxidative stress, suggests membrane damage and nucleic acid impairment, key contributors to neurodegeneration.^20^ The changes in the amide I and II bands observed in the ATR-FTIR spectra (1750-1200 cm^−1^) may also reflect the impact of advanced glycation end products on protein folding and stability, particularly in the formation of toxic aggregates.^21^ Our findings highlight that these spectral features may play a significant role in early detection and monitoring of neurodegenerative processes.

In our study, all six AD spectral digital biomarkers (1358 cm^−1^, 1396 cm^−1^, 1547 cm^−1^, 1628 cm^−1^, 1636 cm^−1^, and 1649 cm^−1^) belong to the 1750-1200 cm^−1^ region, where their intensities consistently showed a decreasing trend in AD patients. For instance, The peak at 1358 cm^−1^ was attributed to the vibration of nucleic acid phosphate,^22^ may reflect phosphate loss due to increased reactive oxygen species (ROS) levels in AD.^23^ The peak at 1396 cm^−1^, related to phospholipids, also decreased, likely due to membrane damage caused by oxidative stress.^24^ The amide I and II peaks (1547 cm^−1^, 1628 cm^−1^, 1636 cm^−1^, and 1649 cm^−1^) showed reduced intensity, possibly correlating with decreased plasma Aβ42 levels,^25^ as protein secondary structures influence spectral band intensity and shape.

Growing evidence supports the utility of ATR-FTIR spectral biomarkers as diagnostic tools for AD.^26^, ^27^, ^28^, ^29^, ^30^ Paraskevaidi et al. showed that ATR-FTIR detection of plasma samples could identify AD with 70% sensitivity and specificity.^28^ Soares et al. found that ATR-FTIR detection based on serum and serum exosomes can be used for the diagnosis of AD.^27^ Our study, however, demonstrated superior performance, with an AUC of 0.92, which was validated in an independent cohort. This improvement may be attributed to our selection of spectral biomarkers based on purified pathological markers, offering higher diagnostic sensitivity and stability. Plasma biomarkers, like p-tau217, Aβ42, and GFAP, have been widely studied for AD diagnostics,^31^ and the spectra of purified biomarkers have been used to derive spectral reference libraries for traumatic brain injury in a previous study.^32^ The spectroscopic fingerprints of these biomarkers provide additional layers of diagnostic information, improving classification accuracy.

However, whether spectral biomarkers panel can be used to identify MCI has not yet been reported. Our study also explored the potential of spectral biomarkers for diagnosing MCI, a precursor to AD. We demonstrated that the model distinguished MCI from HC with an AUC of 0.89 and from AD with an AUC of 0.86, confirming the utility of spectral panels for early diagnosis. The decreasing absorbance of spectral biomarkers correlated with cognitive decline, suggesting that these markers could be useful in monitoring disease progression.

Few studies have explored the ability of spectral biomarkers to differentiate AD from other types of dementia. Only one study by Paraskevaidi et al., including 34 DLB patients and 30 FTD patients, showed that ATR-FTIR spectra biomarkers have a good effect on distinguishing AD from DLB and FTD.^28^ In their study, 1524 cm^−1^ and 1558 cm^−1^ were used to differentiate DLB from AD, whereas 1528 cm^−1^ was used to differentiate FTD from AD.^28^ This is consistent with our findings that seven spectral biomarkers that differentiate DLB from AD and six spectral biomarkers that differentiate FTD from AD were between 1500 and 1560 cm^−1^. This spectral region belongs to the amide II bands, which is consistent with the presence of Aβ fibrils.^19^ Our findings show that this spectral region is important for the differential diagnosis of AD. In this study, we included a large sample of different neurodegenerative diseases and found that panels containing relevant spectral biomarkers achieved good classification results, with AUCs of 0.83 for DLB, 0.80 for FTD, and 0.81 for PSP, suggesting that composite spectral biomarkers can aid in the differential diagnosis of neurodegenerative dementia.

Our study is the first to construct AD spectral biomarkers based on purified pathological markers. The plasma biomarkers p-tau217, GFAP, and Aβ42 contribute to the changes observed in the spectra of AD patients. Furthermore, our results showed that plasma p-tau217 and GFAP levels were negatively correlated with AD spectral biomarkers. As previously discussed, the peaks at 1358 cm^−1^ and 1396 cm^−1^ are related to nucleic acids and phospholipids, respectively, and their intensities may decrease due to increased levels of oxidative stress. Previous studies have shown that p-tau can increase oxidative stress levels.^33^ GFAP, a neuroinflammation marker, is closely associated with oxidative stress.^34^ Therefore, we speculate that increased plasma p-tau217 and GFAP levels mediate the reduction of nucleic acids and phospholipids through oxidative stress, resulting in a decrease in the absorption peaks at 1358 cm^−1^ and 1396 cm^−1^. This correlation provides additional insight into the molecular changes associated with AD and highlights the potential for spectral biomarkers to detect disease-related pathological changes.

In the context of machine learning, model interpretability is crucial for understanding the relationship between spectral biomarkers and AD pathology.^9^ By integrating biomarkers related to Aβ, p-tau, and GFAP, our model allows for transparent interpretation of spectral changes in the context of disease progression. This interpretability is essential for translating these findings into clinical practice, helping clinicians understand the diagnostic significance of spectral biomarkers.

Despite the potential of ATR-FTIR, several limitations exist. Water absorption in the mid-infrared region can obscure spectral information, necessitating sample drying before analysis. Current techniques, such as air drying, are effective but time-consuming, limiting their practicality in clinical settings. Our device addresses this by introducing a rapid drying method, ensuring measurement consistency with an auto-presser that applies uniform contact pressure. This innovation enhances the reproducibility of spectral data, supporting batch-oriented clinical diagnostics.

This study presents three key innovations: Firstly, we construct spectral biomarker panels with high sensitivity and specificity, providing non-invasive insights into AD pathology; Secondly, we developed transparent diagnostic models by linking core pathological AD biomarkers with spectral biomarkers, enhancing clinical applicability; Finally, we introduced a new detection mechanism that improves water removal and measurement consistency, supporting rapid clinical detection.

However, some limitations remain. Firstly, we used synthetic purified proteins to screen for AD biomarkers, which may differ from human pathological proteins. Human pathological proteins are relatively complex in structure, and synthetic purified proteins may only partially reflect the structural characteristics of pathological proteins in humans, potentially limiting their ability to fully represent them. However, it is worth noting that changes in the secondary structure of synthetic Aβ oligomers, as detected by infrared spectroscopy, have been used to elucidate the process of Aβ aggregation.^35^ This suggests that infrared spectra of synthetic Aβ may reflect the secondary structure of Aβ in patients. Although there is currently no literature on spectra of synthetic P-tau and GFAP, we believe that using the spectra of synthetic proteins as a proxy for human pathological proteins is reasonable to some extent. In future studies, we plan to compare the structures of synthetic proteins, especially synthetic P-tau and GFAP, with human pathological proteins to further investigate whether significant differences exist. Secondly, the spectral peaks for differentiating other dementias were distinct from those for AD, as AD-specific biomarkers were selected from a cohort comprising AD and HC participants. AD co-pathology in other dementias, such as elevated p-tau and GFAP levels in FTD and DLB, may account for these differences. For example, p-tau and GFAP levels were found to be increased in FTD and DLB.^36^ Therefore, when distinguishing AD from different types of dementia, the included spectral peaks were different from those used to distinguish AD from HC. Although our study includes the largest sample size in this area of research, covering a wide range of disease types, which provides an advantage in diagnostic and differential diagnosis, there is still room to expand the sample size to further support our model’s findings. Regarding generalizability, we used an independent community cohort to validate the model results. In future work, we plan to include additional external cohorts to independently validate the results of our models.

In conclusion, this study provides a new method for AD diagnosis using blood-based spectral biomarkers panels. The diagnostic model based on composite spectral biomarkers accurately identified AD and MCI in the cohort. Additionally, we found that spectral biomarkers could distinguish patients with AD from those with DLB, FTD, and PSP. Therefore, we believe that plasma spectral biomarkers panels have the potential to be widely used for the diagnosis of AD and other neurodegenerative diseases.

## Contributors

Lu Shen and Zhigang Li had full access to all of the data in the study and took responsibility for the integrity of the data and the accuracy of the data analysis. Lu Shen, Bin Jiao, Zhigang Li and Beisha Tang designed research; Ziyu Ouyang, Xuewen Xiao, Cong Zhang, Tianyan Xu, Qijie Yang, Yuan Zhu, Xixi Liu, Yafang Zhou, Xinxin Liao, and Shilin Luo completed the cognitive assessment and plasma sample collection; Zhigang Li and Ziyu Ouyang performed the research on spectral digital biomarkers; Bin Jiao, Zhigang Li and Ziyu Ouyang analyzed data; Bin Jiao, Zhigang Li and Ziyu Ouyang wrote the paper; Lu Shen supervised the completion of this study.

## Data sharing statement

The data used in this study contains sensitive patient information and cannot be shared publicly but may be made available upon request.

## Declaration of interests

All authors declare no financial or non-financial competing interests.
