# Supplementary material for: Development and validation of machine learning models with blood-based digital biomarkers for Alzheimer’s disease diagnosis: a multicohort diagnostic study
Source: eClinicalMedicine. 2025 Mar 5;81:103142. doi: 10.1016/j.eclinm.2025.103142 (PMC11925590; doi:10.1016/j.eclinm.2025.103142)
Supplement: Supplementary Fig. S1 and Tables S1–S4 [file mmc1.docx]

**Development and Validation of Machine Learning Models with Blood-Based Digital Biomarkers for Alzheimer’s Disease Diagnosis: A Multicohort Diagnostic Study**

***Supplementary information***

This file contains descriptions of the 5 supplementary materials to accompany the main article.

**Table S1…………………………………………………………………………………………………………………………………….……….2**

**Table S2…………………………………………………………………………………………………………………………………….……….3**

**Table S3…………………………………………………………………………………………………………………………………….……….4**

**Table S4…………………………………………………………………………………………………………………………………….……….5**

**Figure S1…………………………………………………………………………………………………………………………………….………6**

Table S1. Comparison of demographics and cognitive levels between patients with MCI, DLB, FTD, PSP and HC in Cohort 1.

|  | HC in Cohort 1  (n = 189) | MCI  (n = 151) | | DLB  (n = 106) | | FTD  (n = 106) | | | PSP  (n = 135) | |
| --- | --- | --- | --- | --- | --- | --- | --- | --- | --- | --- |
|  |  |  | *p* |  | *p* | |  | *p* |  | *p* |
| Age, years | 68 [65-71] | 67 [61-72] | 0.1 | 72 [66-78] | <0.01 | | 63 [55-69] | <0.01 | 66 [60-70] | <0.01 |
| Sex, Male | 88 (46.6) | 71 (47) | 0.9 | 63 (59.4) | <0.01 | | 55 (51.9) | 0.3 | 88 (65.2) | <0.05 |
| ADO, years | - | 63 [57-68] | - | 69.5 [62-75] | - | | 58 [53-66] | - | 63 [57-68] | - |
| COD, years | - | 2 [1-3] | - | 2 [1-4] | - | | 2 [1-4] | - | 2 [1.5-4] | - |
| Education, years | 9 [6-12] | 9 [6-12] | 0.7 | 9 [6-12] | 0.7 | | 9 [6-12] | 0.3 | 9 [6-12] | 0.4 |
| MMSE | 29 [29-30] | 25 [22-27] | <0.01 | 15 [7-22] | <0.01 | | 16 [8-22] | <0.01 | 24 [18-27] | <0.01 |
| MoCA | - | 20 [16-21] | - | 7 [3-13] | - | | 10 [4-15] | - | 14 [10-19] | - |
| CDR | - | 0.5 [0-0.5] | - | 2 [1-2] | - | | 1 [0.5-2] | - | - | - |
| Plasma p-tau217 pg/ml | 4.48 [3.6-6.05] | 3.5 [3-4.01] | <0.01 | - | - | | - | - | - | - |
| Plasma Aβ42 pg/ml | 70.13 [52.11-92.7] | 69.05 [47.74-98.18] | 0.8 | - | - | | - | - | - | - |
| Plasma GFAP pg/ml | 19.15 [14.92-27.91] | 24.67 [16.62-41.5] | <0.01 | - | - | | - | - | - | - |

Data are median [interquartile range] or n (%). ADO, Age of disease onset; COD: course of disease; MMSE, Mini-Mental State Examination; MoCA, Montreal Cognitive Assessment; CDR, Clinical Dementia Rating Scale; HC, healthy control; MCI, mild cognitive impairment; DLB, Lewy Body dementia; FTD, frontal-temporal dementia; PSP, progressive supranuclear palsy; GFAP, glial fibrillary acidic protein.

Table S2. Core spectra digital biomarkers and machine learning-based spectral features in this study.

| Classification | Spectral digital biomarker (cm^-1^) | Number |
| --- | --- | --- |
| Core spectra digital biomarkers | 1329, 1358, 1396, 1409, 1452, 1456, 1503, 1547, 1622, 1628, 1636, 1649, 2852, 2885, 2926, 2936, 3182, 3260, 3274, 3288 | 20 |
| Machine learning-based spectral features | 1126, 1138, 1192, 1276, 1340, 1358, 1396, 1416, 1430, 1472, 1490, 1497, 1547, 1554, 1560, 1576, 1582, 1588, 1628, 1636, 1642, 1649, 1655, 1678, 1682, 1684, 1686 | 27 |

Table S3. Biochemical assignments for the twenty-seven wavenumbers selected by the machine learning model.

| Machine learning-based spectral biomarkers (cm^-1^) | Biochemical assignments |
| --- | --- |
| 1126 | ν(C–O), disaccharides, sucrose |
| 1138 | Oligosaccharide C–OH stretching band; 2-methylmannoside |
| 1192 | Phospholipids |
| 1276 | Vibrational modes of collagen proteins-amide III |
| 1340 | CH_2_ wagging; Collagen |
| 1358 | Stretching C–O, deformation C–H, deformation N–H |
| 1396 | Symmetric CH_3_ bending of the methyl groups of proteins |
| 1416 | Deformation C–H, N–H, stretching C–N |
| 1430 | δ(CH_2_) (polysaccharides, cellulose) |
| 1472 | CH_2_ bending of the methylene chains in lipids |
| 1490 | C = C, deformation C–H ;In-plane CH bending vibration |
| 1497 | C= C, deformation C–H |
| 1547 | Amide II: (protein N–H bending, C–N stretching), a-helical structure |
| 1554 | Predominately a-sheet of amide II (amide II band mainly stems from the C–N stretching and C–N–H bending vibrations weakly coupled to the CHO stretching mode) |
| 1560 | Ring base |
| 1576 | C=N adenine ; Thymine |
| 1582 | Ring C–C stretch of phenyl |
| 1588 | C=C stretching of quinoid rings |
| 1628 | Amide I region |
| 1636 | C=O stretching of carbonyl group, typical saccharide absorption |
| 1642 | Amide I band (arises from C=O stretching vibrations) |
| 1649 | Unordered random coils and turns of amide I |
| 1655 | Amide I (of proteins in a-helix conformation) |
| 1678 | Unordered random coils and turns of amide I |
| 1682 | Adenine |
| 1684 | C=O guanine deformation N–H in plane |
| 1686 | Amide I β-turns of proteins |

Table S4. Comparison of the performance of six machine learning classification models.

| Methods |  | Validation set | |  | Testing set | |
| --- | --- | --- | --- | --- | --- | --- |
|  |  | Sensitivity | Specificity |  | Sensitivity | Specificity |
| Random Forest (RF) |  | **91.6%** | **83.3%** |  | **88.2%** | **84.1%** |
| Support Vector Machine (SVM) |  | 85.0% | 88.3% |  | 83.8% | 81.8% |
| K-Nearest Neighbors (KNN) |  | 81.6% | 88.3% |  | 80.8% | 83.3% |
| Logistic Regression (LR) |  | 86.6% | 83.3% |  | 83.8% | 84.7% |
| Linear discriminant analysis (LDA) |  | 88.3% | 81.6% |  | 83.8% | 82.6% |
| Back Propagation Neural Network (BP Neural Network) |  | 76.5% | 87.7% |  | 73.3% | 90.0% |

**Figure S1.** FTIR spectroscopy test platform with auto contact ATR.
